# Variable-Damping Impedance Control for Contact Tasks: A Reinforcement Learning Method Integrating HER and Importance Sampling

**DOI:** 10.3390/s26144538

**Published:** 2026-07-17

**Authors:** Xiaoqiang Guo, Hongchang Ding, Xin Ning, Han Hou, Jinhua Cai

**Affiliations:** 1 School of Mechatronic Engineering, Changchun University of Science and Technology, Changchun 130022, China; 2024200122@mails.cust.edu.cn (X.G.); 2023200103@mails.cust.edu.cn (X.N.); 2023200111@mails.cust.edu.cn (J.C.); 2 Chongqing Research Institute, Changchun University of Science and Technology, Chongqing 401133, China; eric_houyz@163.com

**Keywords:** deep reinforcement learning, impedance control, industrial robot, contact force tracking

## Abstract

Force control is crucial for robotic contact-rich tasks such as assembly, grinding, and polishing, directly affecting task accuracy, interaction stability, and safety. Yet in unstructured environments, environmental uncertainty, contact oscillations, and friction disturbances make high-performance contact control and reinforcement learning policy optimization difficult. To address this issue, this paper proposes a deep reinforcement learning-based variable-damping impedance control method that integrates parameterized Hindsight Experience Replay (TO-HER) and Importance Sampling (IS). Within the impedance control framework, a residual parameterized policy enables online damping adjustment, improving dynamic adaptability across contact phases. To overcome the training instability of conventional HER in contact-intensive tasks, a parameterized goal relabeling mechanism is introduced to improve relabeled sample quality and sample efficiency. In addition, an importance sampling scheme based on density ratio estimation mitigates the distribution mismatch between relabeled and real samples, enhancing training quality and stability. Experimental results show that the proposed method outperforms baseline methods in convergence, final return, and training stability, while achieving higher tracking accuracy and better dynamic response in typical contact scenarios, demonstrating strong effectiveness and robustness for robotic contact control in unstructured environments.

## 1. Introduction

Robot force control is a fundamental technique in precision manufacturing and intelligent interaction. It has been widely used in tasks such as polishing, assembly, material handling, and human–robot collaboration [1,2,3,4]. These tasks require robots to regulate interaction forces while tracking positions, so as to establish stable and compliant physical contact. However, in unstructured or dynamic environments, the contact establishment process is often accompanied by transient impacts, oscillations, and force-tracking errors, making it difficult to simultaneously ensure contact stability and force-tracking accuracy.

Existing approaches to robotic force regulation mainly include force/position hybrid control and impedance control [5,6]. Force/position hybrid control relies on explicit task-space decomposition and usually performs well when contact constraints are clearly defined. However, its adaptability is often limited when the environment is uncertain or contact conditions change abruptly. Impedance control, by contrast, regulates the interaction through a dynamic relationship between the end-effector motion and the contact force, and therefore provides an effective framework for compliant interaction [7,8]. Nevertheless, conventional impedance control usually employs fixed parameters. When environmental stiffness, contact boundaries, or external disturbances vary, fixed-parameter designs may fail to maintain consistent force-tracking performance and contact stability [9].

To improve robotic adaptability in complex contact environments, researchers have introduced adaptive control mechanisms into impedance control frameworks [10,11,12]. In parallel, robust and hybrid impedance designs have also been developed to enhance system tolerance to modeling errors and disturbances [13,14,15]. Although these methods improve adaptability to some extent, many of them still depend on carefully designed models or manually tuned rules, which limits their flexibility in complex contact scenarios.

More recently, learning-based methods have attracted increasing attention for online impedance adaptation and force-control optimization [16,17,18]. Neural-network-based approaches have shown the ability to compensate for model uncertainties and external disturbances through nonlinear approximation [19,20]. Representative studies include wavelet-neural-network-based compensation, adaptive Jacobian compensation, and neural-network-assisted admittance or impedance control [21,22,23]. Compared with model-dependent adaptive control, reinforcement learning provides a model-free framework that can optimize control strategies through interaction with the environment [24,25]. It has gradually been introduced into robotic contact tasks for tuning impedance-related parameters, optimizing damping behavior, and improving contact strategies in applications such as assembly, polishing, and space manipulation [26,27,28,29]. Despite this progress, most reinforcement-learning-based methods still require large amounts of interaction data and long training times. For robotic contact tasks, where interactions are complex and data acquisition is costly, this issue significantly restricts practical deployment on real systems.

To alleviate sparse rewards and low sample efficiency in goal-conditioned reinforcement learning, Hindsight Experience Replay (HER) typically improves the utilization of unsuccessful trajectories by relabeling subsequently achieved states as alternative goals, and has therefore been widely used in goal-conditioned reinforcement learning [30,31,32,33]. However, in robotic contact tasks, directly using subsequently achieved states from raw physical trajectories as virtual goals can easily introduce undesirable dynamics such as collision noise, high-frequency oscillations, and sudden local friction variations into the goal relabeling process, thereby reducing the effectiveness of virtual goals and compromising the stability of policy learning. Therefore, designing a goal relabeling strategy suitable for contact tasks is crucial to improving the effectiveness of HER in such scenarios.

To address these limitations, an adaptive variable-damping impedance control strategy is developed in this work based on an enhanced Deep Deterministic Policy Gradient (DDPG) algorithm. The method realizes real-time tuning of the damping parameter under the impedance control framework, which enhances dynamic adaptation across distinct contact phases. Furthermore, a parameterized HER mechanism is introduced to generate virtual goals that are better suited to contact control requirements and to improve sample efficiency. To address the distribution mismatch between virtual samples generated by parameterized goal relabeling and real interaction samples, an importance-weighted update mechanism based on discriminator-driven density-ratio estimation is further designed to improve the stability of value estimation and policy optimization.

The main contributions of this paper are summarized as follows:

1. To address the issue that original HER directly adopts subsequently achieved states for goal relabeling in contact tasks, which may introduce contact noise and undesirable local dynamics, a parameterized HER mechanism is developed to improve the effectiveness of virtual goal construction and the efficiency of sample reuse.
2. To mitigate the distribution mismatch between virtual samples generated by parameterized HER and real interaction samples, an importance-weighted update method based on discriminator-driven density-ratio estimation is designed to improve the stability of value estimation and policy optimization.
3. A residual-based variable-damping impedance control framework for robotic contact tasks is proposed. Within the impedance control model, the damping parameter is adaptively adjusted online, thereby improving the system’s dynamic adaptability during both the contact establishment phase and the stable contact phase.

## 2. Robot–Environment Contact Analysis

### 2.1. Robot–Environment Contact Model

In practical contact tasks, the properties of the environment are usually complex and uncertain. For the sake of analytical simplicity, the environment is modeled as a linear elastic body, where $X_{e}$ denotes its equilibrium position and $K_{e}$ represents its stiffness. After the robot end-effector comes into contact with the environment, the contact force acting on the end-effector can be described by Hooke’s law as

(1) $$F_{e}=\left\{\begin{array}{c} K_{e}(X-X_{e}),\\ 0 \end{array}\right.\begin{array}{c} X>X_{e}\\ X\leq X_{e} \end{array}$$

where X represents the actual position of the robot end-effector.

**Figure 1:**
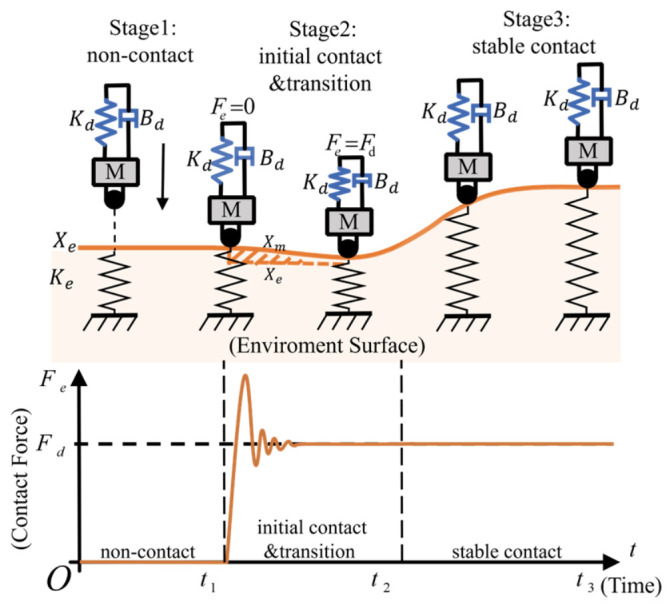
Robot–environment contact model.

As shown in Figure 1, the robot-environment contact process generally consists of three stages: non-contact, initial contact and transition, and stable contact. In Figure 1, M denotes the equivalent mass of the robot end-effector; $K_{d}$ and $B_{d}$ denote the desired stiffness and damping of the impedance model, respectively; $K_{e}$ denotes the environmental stiffness; $X_{e}$ denotes the equilibrium position of the environment; $X_{m}$ denotes the maximum indentation position reached during the initial contact and transition stage; $F_{e}$ and $F_{d}$ denote the actual and desired contact forces, respectively; and $t_{1}$, $t_{2}$ and $t_{3}$ denote the time instants separating the three stages.

During the non-contact stage, the end-effector has not yet interacted with the environment, and the contact force remains zero. During the initial contact and transition stage, the end-effector moves from free space into the constrained environment, which causes a transient abrupt variation in the contact force. In the stable contact stage, after a short adjustment process, the system maintains continuous contact with the environment and performs the desired force-related task under environmental constraints. In the second and third stages, the conventional impedance model of the robot end-effector is given by:

(2) $$M_{d}({\ddot{X}}_{r}-\ddot{X})+B_{d}({\dot{X}}_{r}-\dot{X})+K_{d}(X_{r}-X)=F_{e}-F_{d}$$

where $M_{d}$, $B_{d}$ and $K_{d}$ represent the desired inertia, damping, and stiffness matrices, respectively; $X_{r}$ denotes the reference trajectory of the robot; $F_{d}$ is the desired contact force; and $F_{e}$ is the actual contact force. Since this study focuses on force regulation after contact establishment, the following analysis is mainly concerned with the impedance interaction process from the post-impact stage to the stable-contact stage.

### 2.2. Force-Tracking Error Analysis and Impedance Parameter Decoupling

To reveal the dynamic influence mechanism of impedance parameters on contact force error, the system is analyzed in the Laplace domain. By combining the dynamic equation in Section 2.1 and taking the Laplace transform of Equation (2), the system can be expressed in the s domain as

(3) $$\left(M_{d}s^{2}+B_{d}s+K_{d})(X_{r}(s)-X(s))=F_{e}(s)-F_{d}(s)=\Delta F(s\right)$$

where s is the Laplace operator and ΔF(s) denotes the force-tracking error. If the environment is modeled as a linear stiffness model, the environmental contact force satisfies

(4) $$F_{e}(s)=K_{e}(X(s)-X_{e}(s))$$

Accordingly, the relationship among the actual end-effector position X(s), the environmental position $X_{e}(s)$, and the environmental contact force $F_{e}(s)$ can be obtained as

(5) $$X(s)=X_{e}(s)+\frac{F_{e}(s)}{K_{e}}$$

Substituting Equation (5) into Equation (3) yields the closed-loop relationship of the contact force error:

(6) $$\Delta F(s)(1+\frac{M_{d}s^{2}+B_{d}s+K_{d}}{K_{e}})=(M_{d}s^{2}+B_{d}s+K_{d})(X_{r}(s)-X_{e}(s))-F_{d}(s)$$

where $Z(s)=M_{d}s^{2}+B_{d}s+K_{d}$ represents the desired impedance model of the robot. By further rearranging, the force error transfer function can be written as

(7) $$\Delta F(s)=\frac{K_{e}Z(s)}{K_{e}+Z(s)}(X_{r}(s)-X_{e}(s))+\frac{Z(s)}{K_{d}+Z(s)}F_{d}(s)$$

It can be inferred from Equation (7) that the contact force error depends not only on the impedance parameters $M_{d}$, $B_{d}$, $K_{d}$, but also on the environmental stiffness $K_{e}$ and the environmental position $X_{e}(s)$.

To further investigate the steady-state behavior of the system, the steady-state error as t→∞ can be expressed as:

(8) $$\Delta f_{ss}=\underset{s\to 0}{\lim}s\cdot \frac{\Delta F(s)}{s}=\underset{s\to 0}{\lim}\Delta F(s)$$

Under a step reference input, the steady-state error can be further obtained as

(9) $$\Delta f_{ss}=\frac{K_{e}K_{d}}{K_{e}+K_{d}}(x_{r}-x_{e})+\frac{K_{e}}{K_{e}+K_{d}}f_{d}$$

Equation (9) indicates that, if the environmental stiffness $K_{e}$ and equilibrium position $x_{e}$ can be accurately estimated and compensated, the steady-state force-tracking error can theoretically converge to zero. However, in practical contact tasks, environmental stiffness and position are usually difficult to measure and estimate online with high accuracy. Therefore, this paper adopts the simplified strategy of setting $K_{d}\approx 0$, thereby shifting the focus of impedance regulation from the stiffness channel to the damping channel so as to avoid dependence on accurate identification of environmental parameters.

Consequently, the contact force error is jointly affected by the desired impedance parameters and the unknown environmental characteristics. Since the environmental stiffness and contact position are difficult to identify accurately in real applications, conventional fixed-parameter impedance control can hardly maintain both stable and accurate force regulation in unstructured contact scenarios. To address this issue, a goal-conditioned Markov decision process is formulated along the dominant contact axis, and the online adaptive adjustment of the damping parameter is subsequently investigated.

## 3. Variable Impedance Control Method Based on DRL-HER

**Figure 2:**
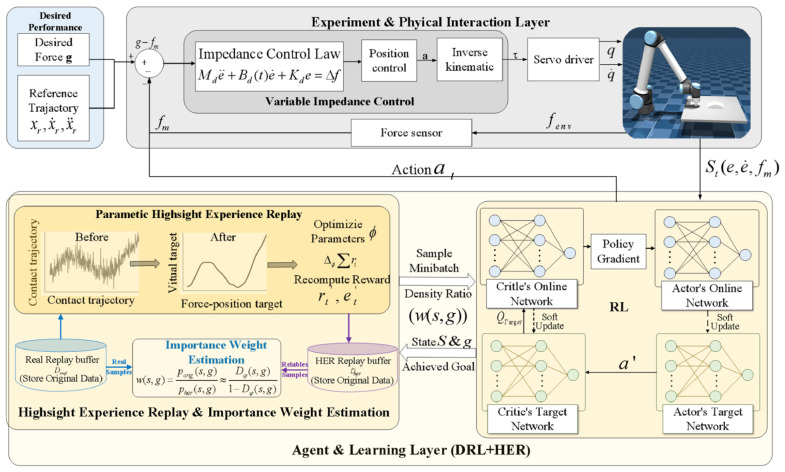
Reinforcement-learning-based intelligent optimization framework.

This paper presents a distribution-shift-corrected variable impedance control algorithm based on parameterized HER for unstructured contact tasks, in which importance sampling is incorporated to reduce the distribution mismatch of relabeled samples and enhance both training stability and force control performance. The overall reinforcement-learning-based intelligent optimization framework is shown in Figure 2.

### 3.1. General Markov Decision Process Formulation for Contact Tasks

Considering that practical contact interactions usually occur mainly along a single normal dominant axis, and that reinforcement learning methods often suffer from high sample complexity and unstable training in high-dimensional state spaces, the contact control problem is reduced to a one-dimensional dominant contact axis for modeling. The standard one-dimensional impedance relationship of the robot end-effector in Cartesian space can be expressed as:

(10) $$m_{d}({\ddot{x}}_{r}-\ddot{x})+b_{d}(t)({\dot{x}}_{r}-\dot{x})+k_{d}(x_{r}-x)=-f_{e}$$

where $m_{d}$, $b_{d}(t)$, and $k_{d}$ denote the desired inertia, time-varying damping, and desired stiffness parameters, respectively, with $m_{d},b_{d}(t),k_{d}\in {\mathbb{R}}^{+}$; $x,x_{r}\in \mathbb{R}$ denote the actual end-effector position and the reference position, respectively.

When the desired stiffness term approaches zero, the system dynamics can be further simplified into the one-dimensional error dynamics:

(11) $$m_{d}\ddot{e}+b_{d}(t)\dot{e}=\Delta f$$

To balance safety in the early stage of training and the achievable performance upper bound in the later stage, the time-varying damping is decomposed into a fixed base term and a variable residual term, i.e.,

(12) $$b_{d}(t)=b_{base}+\alpha \cdot \Delta b_{\pi }$$

where $b_{base}>0$ is the base damping, $\Delta b_{\pi }$ is the variable residual term, and α is a scaling factor. The task is formulated as a goal-conditioned Markov decision process. In the one-dimensional single-axis contact task, the system state is defined as

(13) $$s_{t}={\left[x_{c,t},{\dot{x}}_{c,t},f_{e,t},a_{t-1},g_{t}\right]}^{T}\in {\mathbb{R}}^{5}$$

which includes position information, contact force information, error information, the previous action, and the current goal variable. To achieve online adaptation of the low-level controller, the continuous action of the agent is directly mapped to the damping residual term in Equation (12), i.e., the action is defined as

(14) $$a_{t}=\Delta b_{\pi }(a_{t}\geq 0)$$

To simultaneously account for tracking accuracy and control smoothness, a reward function $R_{t+1}$ composed of an error term and an action-smoothing term is designed as

(15) $$\begin{array}{l} R_{t+1}=r_{err}+r_{smo}\\ r_{err}=\lambda_{1}e^{-\frac{{\left|e\right|}^{2}}{\sigma_{x}^{2}}}+\lambda_{2}e^{-\frac{{\left|\Delta f\right|}^{2}}{\sigma_{f}^{2}}}\\ r_{smo}=-\lambda_{3}{\left|a_{t}-a_{t-1}\right|}^{2} \end{array}$$

where $\lambda_{1}$, $\lambda_{2}$ and $\lambda_{3}$ are the weighting coefficients of each term. The term $r_{err}$ is used to penalize the contact force error and position error. $\sigma_{x}$ and $\sigma_{f}$ are Gaussian kernel parameters introduced to provide smoother gradient variations. The term $r_{smo}$ is used to suppress abrupt changes in the damping parameter between adjacent time steps.

### 3.2. Parameterized Hindsight Goal Relabeling Mechanism and Distribution Shift Correction

In continuous surface contact tasks, conventional reinforcement learning methods still suffer from low exploration efficiency and sparse effective samples. Hindsight Experience Replay (HER) usually improves sample utilization by relabeling posterior states in a trajectory as substitute goals. However, if the posterior states of the original physical trajectory are directly used as virtual goals, collision noise, high-frequency oscillations, and local abrupt friction variations may be introduced into the learning process, thereby reducing the effectiveness of goal relabeling.

To address this issue, a parameterized virtual goal generation mechanism is adopted to smoothly reconstruct the goal sequence within a local temporal window. For the one-dimensional dominant contact axis, the desired position trajectory and desired contact force can be respectively expressed as

(16) $$\begin{array}{l} X_{d}^{\prime }(t;\theta_{x})=c_{0}+c_{1}t+c_{2}t^{2}+c_{3}t^{3}\\ F_{d}^{\prime }(t;\theta_{f})=f_{d}+\Delta f_{e}e^{-\alpha t} \end{array}$$

where the position parameter vector is defined as $\theta_{x}=\{c_{0},c_{1},c_{2},c_{3}\}\in {\mathbb{R}}^{4}$ and the force-goal parameter vector is defined as $\theta_{f}=\{f_{d},\Delta f_{e},\alpha \}\in {\mathbb{R}}^{3}$. Then the complete parameter set is denoted by $\theta =\{\theta_{x},\theta_{f}\}$.

To make the generated virtual goal trajectory as close as possible to the real physical interaction process in terms of reward, the goal relabeling process is formulated as a cumulative reward maximization problem within a local time window:

(17) $$J(\theta )=\sum_{t=0}^{H}r({X^{\prime }}_{d}(t;\theta_{x})-X_{env}(t),\;{F^{\prime }}_{d}(t;\theta_{f})-F_{env}(t))$$

where H is the local temporal window. Since both the virtual trajectory generation function and the reward function are continuously differentiable with respect to θ over the real domain, a first-order gradient ascent method is adopted:

(18) $$\theta_{k+1}=\theta_{k}+\alpha \nabla_{\theta }J(\theta_{k})$$

where α is the step size. After a small number of iterations, the optimization converges to a local optimum $\theta^{*}$, based on which a smooth virtual goal sequence can be generated. After goal replacement, the system recomputes the state error and immediate reward according to the new virtual goal, and stores the relabeled transition samples into the HER buffer.

### 3.3. Distribution Shift Correction with Adaptive Importance Sampling

Although parameterized goal relabeling improves the learnability of samples, it also introduces a distribution shift problem. In the Actor-Critic reinforcement learning framework, the original physical interaction samples follow the true joint state-goal distribution $p_{orig}(s,g)$. In contrast, the samples relabeled by parameterized HER follow another distribution $p_{her}(s,g)$, which often deviates from the true physical exploration distribution. If this mismatch is ignored and the relabeled samples are directly used to update the network parameters, value estimation bias and distorted policy updates may occur, thereby affecting training stability.

To reduce the above bias, an adaptive importance sampling mechanism based on density-ratio estimation is introduced. For a relabeled sample (s,a,g), the correction weight is defined on its associated state-goal pair (s,g) as the density ratio between the original distribution and the relabeled distribution:

(19) $$w(s,g)=\frac{p_{orig}(s,g)}{p_{her}(s,g)}$$

This weight reflects the reliability of a relabeled sample under the true physical interaction distribution. Based on this, the corrected loss function of the Critic network can be written as

(20) $$L_{Q}(\phi )=E_{(s,a,g)\sim p_{her}}\left[w(s,g)\cdot {\left(Q_{\phi }(s,a,g)-y_{t}\right)}^{2}\right]$$

where s, a, and g denote the state, action, and goal, respectively; $Q_{\phi }(s,a,g)$ is the Critic network parameterized by ϕ; $p_{her}$ denotes the distribution of relabeled samples in the replay buffer; and $y_{t}$ is the temporal-difference target computed by the target network.

Since the state space in contact control tasks contains multidimensional continuous information, it is difficult to explicitly estimate the probability densities $p_{orig}(s,g)$ and $p_{her}(s,g)$. Therefore, a discriminator network $D_{\psi }(s,g)$ is introduced to distinguish whether a sample comes from the original replay buffer or the relabeled replay buffer.

Its training objective and the corresponding density-ratio estimation are given by

(21) $$L_{D}(\psi )=E_{(s,g)\sim p_{orig}}[-\log D_{\psi }(s,g)]+E_{(s,g)\sim p_{her}}[-\log(1-D_{\psi }(s,g))]$$

(22) $$w_{\psi }(s,g)=\frac{D_{\psi }^{*}(s,g)}{1-D_{\psi }^{*}(s,g)}$$

After obtaining the weight $w_{\psi }(s,g)$, it is incorporated into the deterministic policy gradient update of the Actor network:

(23) $$\nabla_{\phi }J(\phi )=E_{(s,g)\sim D}\left[w(s,g)\cdot \frac{\partial Q_{\omega }(s,g,a)}{\partial a}{|}_{a=\mu_{\phi }(s)}\cdot \nabla_{\phi }\mu_{\phi }(s)\right]$$

In this way, the update bias caused by distribution shift can be alleviated during policy learning, thereby improving training stability. For the target networks of both the Critic and the Actor, soft updates are performed using exponential moving averages.

Based on the above design, the overall training procedure is summarized in Algorithm 1.
**Algorithm 1:** Parameterized DRL-HER Variable-Damping Control Algorithm1. Initialize the Actor, Critic, discriminator, and their target networks2. Initialize the replay buffers $D_{orig},D_{her}$3. for episode=1,2,…,M do4.      Obtain the initial state $s_{0}$ and the desired goal g5.      for t=1,2,…,T step do6.           Select an action: $a_{t}=\max(\mu_{\phi }(s_{t})+N,0)$7.           Obtain the next state $s_{t+1}$ and reward $r_{t}$8.           store the relabeled samples into $D_{orig}$9.      end for10.     Optimize the parameter $\theta^{*}$ based on the current trajectory,11.     generate virtual goals, construct HER samples, and store them into $D_{her}$12.     for j=1,2,…,N do13.          Sample mini-batches from $D_{orig}$ and $D_{her}$, respectively.14.          Update the discriminator network and compute the density-ratio weight:                                                $w(s,g)=D_{\psi }/(1-D_{\psi })$15.          Update the Critic network $\theta^{q}$ using the weighted TD error16.          Update the Actor network $\theta^{\mu }$ using the weighted policy gradient17.          Soft-update the target networks $\theta^{q}$ and $\theta^{\mu }$18.     end for19.end for

## 4. Simulation Analysis and Comparative Experiments

### 4.1. Simulation Analysis

To satisfy the demands of contact operations in unknown environments, this study concentrates on compliant force control along the principal force direction, namely the Z-axis in the Cartesian coordinate system, and develops a low-level control framework based on single-axis impedance dynamics. To maximize the absorption of initial collision impact and ensure compliant surface conformity of the manipulator end-effector, the nominal stiffness along the Z-axis is set to $K_{base,z}=0$, and the nominal damping is set to $B_{base,z}=50$. The policy network outputs a continuous action command $a_{t}\in [-1,1]$, which is mapped to the dynamic residual adjustment of the Z-axis damping as

(24) $$B_{d}(t)=B_{base,z}+\frac{\left(a_{t}+1\right)}{2}B_{range}$$

where the dynamic adjustment range is set to $B_{range}=130\mathrm{Ns}/m$. Therefore, the actual physical damping varies within [50,180].

#### 4.1.1. MuJoCo-Based Simulation Platform

**Figure 3:**
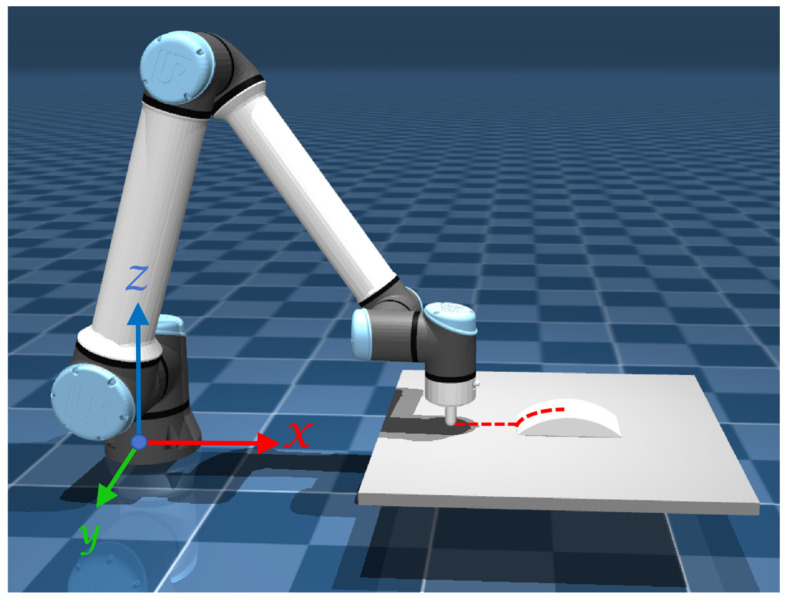
Robot simulation training environment. The red dashed line indicates the motion trajectory of the robot end-effector on the surface.

To verify the effectiveness of the proposed method in unstructured contact environments, a high-fidelity robot contact simulation platform was established in MuJoCo. A simulation model of a six-degree-of-freedom UR10e manipulator was constructed, with a spherical end-effector mounted at the robot tip. A flat platform together with an arc-shaped surface was placed underneath the manipulator to mimic sliding contact on unknown geometric profiles. The corresponding simulation environment is shown in Figure 3.

The Actor network is implemented as a fully connected neural network with two hidden layers of 256 and 128 units, respectively, using ReLU nonlinearities. Its output layer applies Tanh to generate normalized control actions. The Critic network adopts a three-hidden-layer fully connected structure with layer sizes [256, 256, 256], and ReLU is likewise used as the activation function.

The Actor and Critic learning rates are configured as $1\times 10^{-4}$ and $1\times 10^{-3}$, respectively. The replay buffer size is set to $10^{6}$. A soft target update mechanism is employed with smoothing coefficient. To avoid overly rapid discounting, the reward discount factor is set to γ = 0.9995, and the HER ratio k is set to 2. In addition, to balance exploration capability and steady-state accuracy, exponentially decaying Gaussian noise is added to the action output, with an initial standard deviation of 0.2. The truncation length of each training episode is set to 12 s.

#### 4.1.2. Training Performance and Parameter Convergence Analysis

**Figure 4:**
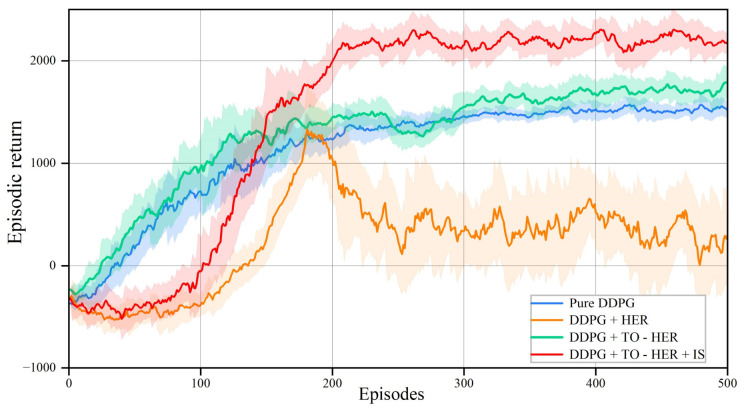
Comparison of training return curves for different methods. Solid curves denote the average episodic return over 10 random seeds; shaded bands indicate ±1 standard deviation.

In the training process, the robot end-effector moves uniformly at 10 mm/s along the X-direction, with the target contact force in the Z-direction set to 20 N, and the total travel distance of the trajectory is 100 mm. The first 50 mm corresponds to a horizontal planar segment with a reference height of Ze = 0.3; the remaining 50 mm is an arc-shaped slope segment, whose horizontal projection length and vertical height are both 50 mm. Figure 4 illustrates the return trajectories of different methods, in which the solid curves denote the average episodic return across 10 random seeds and the shaded bands correspond to ±1 standard deviation.

As shown in Figure 4, although Pure DDPG gradually converges, its convergence speed is relatively slow, and its final return is limited. DDPG + HER exhibits a temporary performance improvement in the middle stage of training, but the return drops significantly in the later stage and shows large fluctuations, indicating that directly incorporating HER introduces considerable instability in this contact task. In contrast, DDPG + TO-HER improves training performance more rapidly and maintains a relatively high return afterward. With the further introduction of importance sampling, DDPG + TO-HER + IS achieves the best performance in terms of convergence speed, final return, and training stability. The quantitative results are summarized in Table 1.

Table 1 further reports the final training performance of different methods. It can be seen that DDPG + TO-HER + IS outperforms all other methods in terms of Final Mean ± SD, Best Return, and Conv. Seeds, achieving a final average return of 2215.7 ± 52.6, with all 10 random seeds successfully converging. These results indicate that parameterized goal relabeling can effectively improve sample efficiency, while distribution-shift correction further enhances training stability and convergence reliability.

#### 4.1.3. Constant-Force Tracking Performance in Unknown Environments

**Figure 5:**
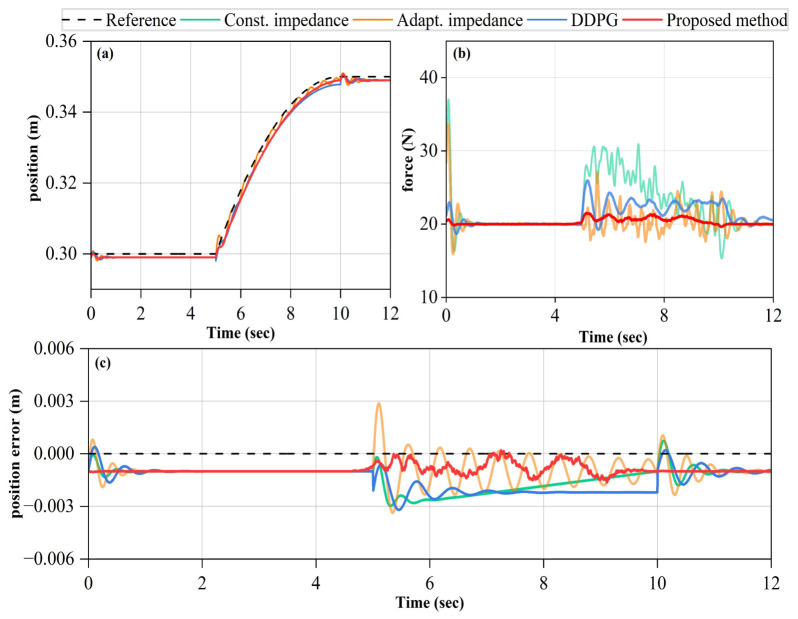
Dynamic responses of different control methods in the arc-surface contact environment: (**a**) position tracking, (**b**) force tracking, and (**c**) position error.

To assess the contact control capability of the proposed approach in unknown and unstructured environments, comparative simulations were performed in an arc-surface contact scenario. The proposed approach was evaluated against constant impedance control, adaptive impedance control, and conventional DDPG, with the target contact force fixed at 20 N for all methods. Figure 5 presents the dynamic responses of the different methods in terms of position tracking, contact force tracking, and position error.

From Figure 5a, it can be observed that all methods can generally follow the reference trajectory at the macroscopic level. However, when entering regions with abrupt geometric changes and stiffness variations, clear differences emerge in compliant adaptability among different control strategies. Combined with Figure 5b, it can be seen that both constant impedance control and conventional DDPG exhibit obvious position deviations, while adaptive impedance control undergoes severe oscillations in the transition region. By contrast, the proposed method maintains a smoother trajectory response and smaller position error during the transition stage.

The contact-force response in Figure 5b further shows that traditional impedance methods are prone to pronounced force fluctuations when facing time-varying stiffness environments. Conventional DDPG improves the performance to some extent, but still suffers from persistent dynamic oscillations. In contrast, the proposed method exhibits smaller transient fluctuations and more stable force-tracking performance throughout the entire contact phase, indicating that it can more effectively coordinate the coupling between position regulation and contact-force control.

**Figure 6:**
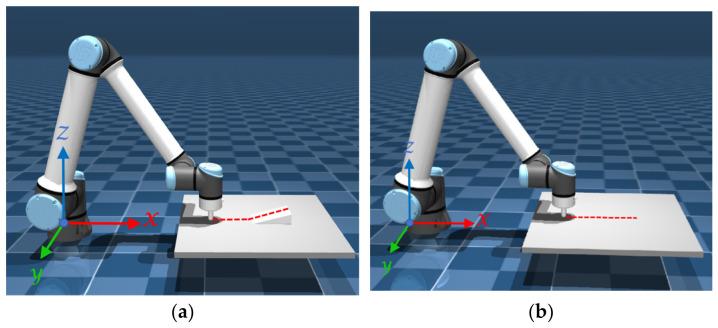
Simulation setups for target contact-force experiments in unknown environments: (**a**) slope-shaped surface and (**b**) flat surface with varying stiffness. The red dashed line indicates the motion trajectory of the robot end-effector on the surface.

To further verify the zero-shot generalization capability of the proposed method, the contact environment was replaced, without retraining the policy network, by a slope-shaped surface and a flat surface with varying stiffness, and the target-force tracking experiments were repeated. The corresponding environments are illustrated in Figure 6.

In a sloped working environment, the robot end-effector is controlled to maintain a constant normal contact force of 20 N against the working surface. The operation trajectory is designed in a segmented manner with a total travel of 100 mm: the first 50 mm constitutes a horizontal planar segment with a reference height set to $z_{e}=0.3$; the remaining 50 mm is an inclined slope segment, which features a horizontal projection length of 50 mm and a vertical height of 80 mm.

**Figure 7:**
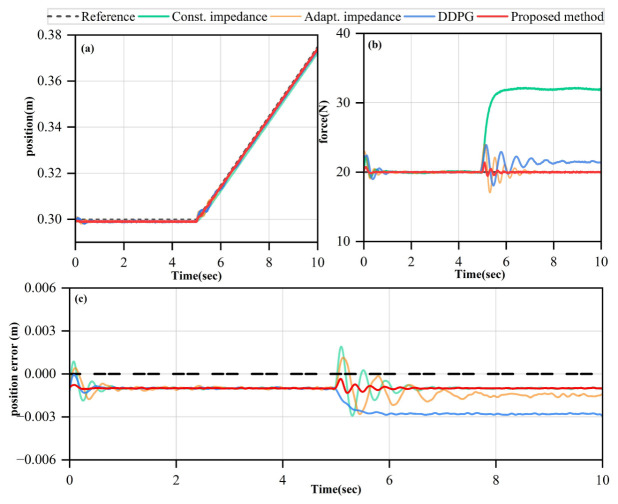
Dynamic responses of different control methods in the slope contact environment: (**a**) position tracking, (**b**) force tracking, and (**c**) position error.

As depicted in Figure 7 for this sloped task scenario, constant impedance control fails to adapt to the continuous variations in surface normal direction and contact stiffness, resulting in a substantial deviation of the contact force from the target value. Although adaptive impedance control and conventional DDPG exhibit a certain degree of adjustment capability, they are still accompanied by large transient overshoot or steady-state error. In contrast, the proposed method achieves smoother position transition and maintains the contact force stably around the target value without introducing any environmental prior information.

**Figure 8:**
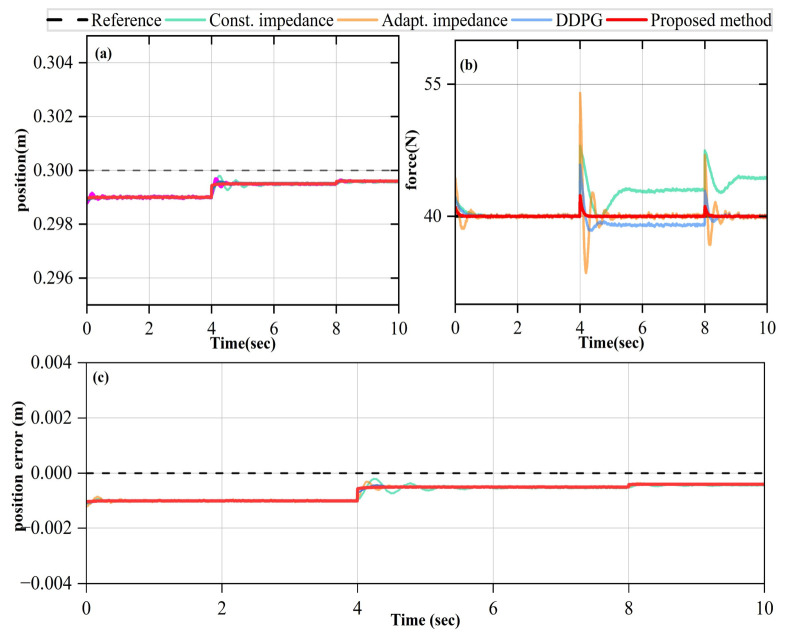
Dynamic responses of different control methods in the varying-stiffness environment: (**a**) position tracking, (**b**) force tracking, and (**c**) position error.

For the varying-stiffness environment, the variable stiffness plane is $z_{e}=0.3$. During the entire interaction process, the target contact force was maintained at 40 N. Figure 8 presents the dynamic responses of different methods under piecewise stiffness changes.

The environmental stiffness is set to:

(25) $$K_{e}=\left\{\begin{array}{c} 2000;\\ 4000;\\ 5000; \end{array}\right.\begin{array}{c} 0s\leq t<4s\\ 4s\leq t<8s\\ 8s\leq t \end{array}$$

The results show that constant impedance control produces obvious steady-state error after abrupt stiffness variation. Conventional DDPG still exhibits persistent bias, while adaptive impedance control is accompanied by large transient impact and oscillations. The proposed method maintains smaller force fluctuation and faster recovery during stiffness transitions, demonstrating better adaptability and robustness against abrupt environmental stiffness changes.

In summary, the simulation results indicate that the proposed method not only improves learning efficiency under limited-sample training conditions, but also translates this advantage into superior contact-control performance in unseen environments, demonstrating favorable constant-force tracking capability and compliant interaction quality under different geometric profiles and stiffness conditions. To further evaluate the practical effectiveness of the learned policy and its sim-to-real transferability, real-world experiments were subsequently conducted on a physical robotic platform.

### 4.2. Experimental Validation of Robot–Environment Interaction

To further investigate the practical effectiveness of the proposed method, a compliant robot–environment interaction platform was established and tested on representative unstructured contact tasks in real-world settings. The policy used in the following experiments was trained entirely in the MuJoCo simulation environment and then directly deployed on the physical UR10e robot after convergence, without any additional fine-tuning or online policy update. During hardware execution, the network parameters remained fixed, and the policy was used only for online inference of the damping adjustment command within the predefined safety range.

**Figure 9:**
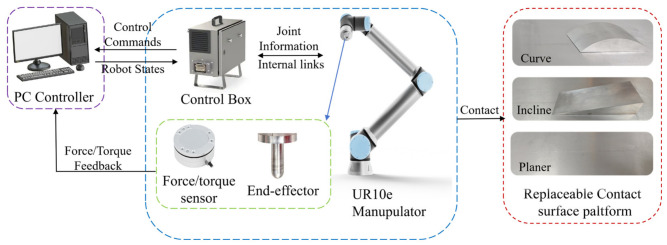
Hardware configuration of the experiment.

**Figure 10:**
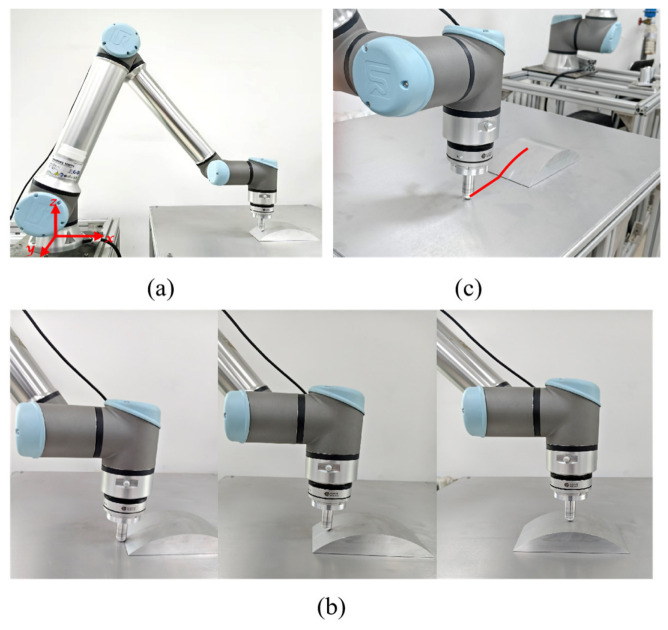
Experimental platform and task configuration for robot–environment interaction: (**a**) overall structure of the experimental system; (**b**) time-series snapshots of the interaction between the end-effector and the contact environment; (**c**) schematic illustration of the reference trajectory and the contact environment.

As illustrated in Figure 9 and Figure 10, the experimental setup primarily includes a host computer, a UR10e robotic manipulator (Universal Robots A/S, Od, Denmark), a six-axis force/torque sensor, and a spherical end-effector. The contact environment is composed of a metal workbench and replaceable contact modules, which were designed to reproduce interaction scenarios with different geometric shapes and constraint conditions. During the experimental trials, the end-effector tracked a predefined reference trajectory, while the controller was responsible for achieving stable regulation of the desired normal contact force. In view of both simulation results and practical safety considerations, constant impedance control, adaptive impedance control, and the proposed method were employed for comparative evaluation.

In terms of system implementation, the host computer is responsible for policy inference and control command transmission, the six-axis force/torque sensor provides real-time measurements of the contact force at the end-effector, and the robot controller performs end-effector trajectory tracking and impedance parameter regulation. Based on this integrated experimental platform, the compliant contact control performance of different methods can be systematically evaluated in real-world scenarios involving continuous curved surfaces, sloped surfaces, and time-varying reference forces.

The hardware experiments were conducted using polished aluminum alloy contact surfaces with a relatively smooth finish. During all tests, the end-effector moved along the x-direction at a constant speed of 10 mm/s over a trajectory length of 300 mm. Three representative contact tasks were designed for validation, including a circular-arc surface, an inclined surface, and a flat surface with a time-varying force reference. The corresponding task-specific geometric settings and reference-force profiles are summarized in Table 2. Since the stiffness and friction properties of the real contact surfaces were not independently calibrated, they were treated as practical environmental uncertainties.

**Figure 11:**
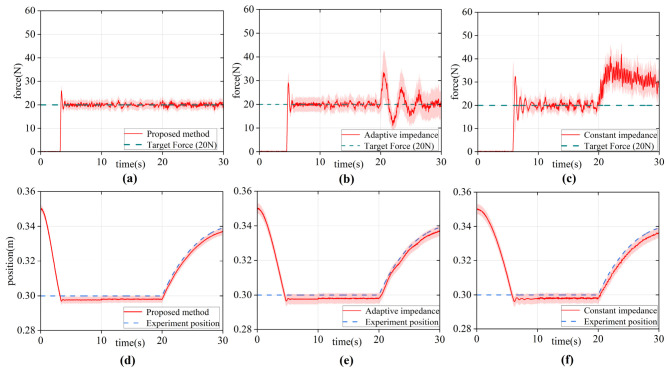
Comparison of dynamic responses of different control methods in the continuous curved-surface contact environment: (**a**) contact-force tracking result of the proposed method; (**b**) contact-force tracking result of adaptive impedance control; (**c**) contact-force tracking result of constant impedance control; (**d**) Z-axis position tracking result of the proposed method; (**e**) Z-axis position tracking result of adaptive impedance control; (**f**) Z-axis position tracking result of constant impedance control. Solid lines represent the mean response over five repeated trials, and the shaded regions indicate mean ± 1 standard deviation.

**Figure 12:**
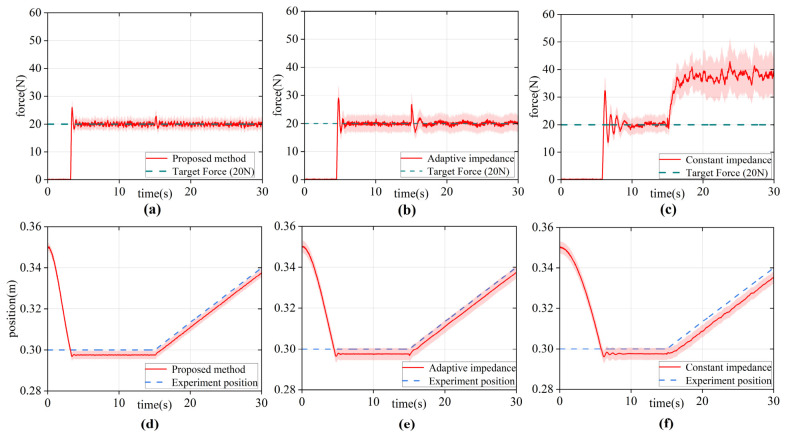
Comparison of dynamic responses of different control methods in the unknown slope contact environment: (**a**) contact-force tracking result of the proposed method; (**b**) contact-force tracking result of adaptive impedance control; (**c**) contact-force tracking result of constant impedance control; (**d**) Z-axis position tracking result of the proposed method; (**e**) Z-axis position tracking result of adaptive impedance control; (**f**) Z-axis position tracking result of constant impedance control. Solid lines represent the mean response over five repeated trials, and the shaded regions indicate mean ± 1 standard deviation.

**Figure 13:**
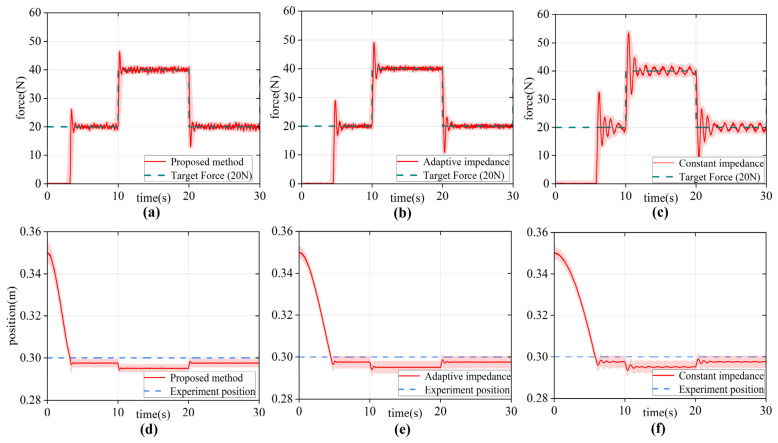
Comparison of dynamic responses of different control methods under varying desired contact force: (**a**) contact-force tracking result of the proposed method; (**b**) contact-force tracking result of adaptive impedance control; (**c**) contact-force tracking result of constant impedance control; (**d**) Z-axis position tracking result of the proposed method; (**e**) Z-axis position tracking result of adaptive impedance control; (**f**) Z-axis position tracking result of constant impedance control. Solid lines represent the mean response over five repeated trials, and the shaded regions indicate mean ± 1 standard deviation.

To ensure a fair comparison, all control methods were evaluated under the same hardware platform and experimental conditions. Each hardware experiment was repeated [n] times under the same controller settings and initial conditions. The force and position trajectories shown in Figure 11, Figure 12 and Figure 13 are presented as the mean response across repeated trials, and the shaded regions denote mean ± 1 standard deviation. The quantitative metrics reported in Table 3 were computed from these repeated experiments to evaluate both tracking accuracy and trial-to-trial consistency.

First, a continuously convex curved surface was selected as the contact object to evaluate the constant-force tracking performance of the controllers in a real nonlinear contact environment, with the target contact force set to 20 N. The corresponding contact force responses and end-effector position trajectories along the Z-axis are presented in Figure 11.

As shown in Figure 11, all three methods are able to track the reference trajectory in repeated hardware trials. However, when interacting with the continuous curved surface, constant impedance control, owing to its fixed parameters, cannot adapt effectively to the time-varying contact constraints, resulting in a noticeable deviation of the contact force from the target value together with relatively large inter-trial variation. Although adaptive impedance control enables online parameter adjustment, evident force fluctuations and dispersion among repeated trials are still observed during the curved-surface transition process. By contrast, the proposed method exhibits a smoother end-effector position response and a narrower variability band, while the contact force converges more stably to approximately 20 N and reaches an essentially steady state at around 4.5 s. These results indicate that the proposed method provides better repeatability and stability under continuously varying contact constraints.

To further evaluate the cross-scenario adaptability of the proposed method in unknown environments, the contact module was replaced with a sloped surface without retraining the policy network, and a constant-force tracking experiment was conducted with the target contact force again set to 20 N. The corresponding results are shown in Figure 12.

Under the unknown sloped-surface condition, the contact force generated by constant impedance control increases with the slope height and finally stabilizes at a value well above the target level, with relatively large variability across repeated trials. Adaptive impedance control can alleviate the steady-state error to some degree, but noticeable overshoot, oscillatory transients, and inter-trial dispersion still remain. In comparison, the proposed method preserves accurate force tracking and smooth positional evolution without policy retraining, while maintaining a comparatively narrower variability range and reaching a near-steady condition at approximately 4.5 s. This result suggests that the proposed method maintains better repeatability, robustness, and cross-scenario generalization in previously unseen environments.

Besides environmental uncertainty, real contact tasks frequently involve variations in the reference force command. For this reason, a further experiment with a time-varying desired contact force was carried out to compare the tracking performance of different controllers under dynamic reference conditions. In this test, the desired contact force changed stepwise at different time instants, and the results are reported in Figure 13.

As shown in Figure 13, when the reference force changes in a stepwise manner, constant impedance control fails to adjust the contact state in a timely manner and exhibits relatively large fluctuations across repeated trials, while adaptive impedance control shows obvious overshoot and oscillatory behavior. In contrast, the proposed method begins to converge at approximately 4.0 s and reaches an essentially steady state at around 4.1 s with comparatively smaller variability. Following the reference-force switch at about 10 s, the peak response of the proposed method is approximately 45.08 N, which is lower than that of adaptive impedance control (49.18 N) and constant impedance control (52.08 N), and the system settles to the new steady state at around 10.32 s. These results suggest that the proposed method exhibits favorable dynamic response, reconvergence behavior, and repeatability under time-varying reference commands.

To provide a quantitative comparison of the control performance of different methods in real contact scenarios, the mean error (Mean Err.), root mean square error (RMSE), and overshoot were computed for the curved-surface contact, sloped-surface contact, and time-varying reference-force cases, as summarized in Table 3.

Table 3 presents the quantitative comparison of the three control methods under curved-surface contact, sloped-surface contact, and time-varying reference-force conditions based on repeated hardware experiments. It can be observed that the proposed method consistently achieves the best overall control performance across all three scenarios. Compared with adaptive impedance control, the proposed method yields the most significant improvement in the curved-surface contact environment, reducing Mean Err. and Overshoot by 70.3% and 60.9%, respectively. In the sloped-surface contact environment, these two metrics are reduced by 54.1% and 35.0%, respectively, while under time-varying reference force conditions, the reductions are 9.0% and 31.8%, respectively. Compared with constant impedance control, the proposed method reduces the Mean Err. across the evaluated scenarios and generally exhibits lower overshoot. These results suggest that the proposed method can help alleviate steady-state force-tracking error and reduce transient fluctuations during contact, thereby improving the smoothness and accuracy of compliant contact control.

## 5. Conclusions

For unstructured robotic contact tasks, this study addresses several key challenges, including significant environmental variability, low sample efficiency, and insufficient training stability in reinforcement learning, by proposing a deep reinforcement learning-based variable-damping impedance control method that integrates parameterized hindsight experience replay with importance sampling. The proposed method constructs a residual variable-damping impedance model along the one-dimensional dominant contact axis, thereby enhancing the robot’s compliant regulation capability during both contact establishment and stable contact phases through online adaptive adjustment of the damping parameter. To overcome the limitations of conventional HER in contact tasks, where directly using posterior states as virtual goals may introduce collision noise, high-frequency oscillations, and local frictional disturbances, a parameterized goal reshaping strategy is developed to improve the quality of relabeled experiences. Furthermore, to mitigate the distribution mismatch between virtual samples and real interaction samples, an importance-weighted update mechanism based on density ratio estimation is incorporated, thereby improving value estimation accuracy and policy optimization stability in the Actor–Critic learning process.

Simulation results demonstrate that the proposed method achieves favorable training convergence and consistency. Specifically, it attains a final average return of 2215.7 ± 52.6 and successfully converges under all 10 out of 10 random seeds. Real-robot experiments further show that, under three operating conditions, namely curved-surface contact, sloped-surface contact, and time-varying reference force tracking, the proposed method achieves mean errors of 0.516 N, 0.311 N, and 0.614 N, respectively, with corresponding overshoots of 26.32%, 29.57%, and 31.06%. Compared with adaptive impedance control, the proposed method shows reductions of up to 70.3% in mean error and 60.9% in overshoot. These results indicate that the method is conducive to improving sample utilization and training stability in reinforcement learning-based contact control, while offering more accurate force tracking and more stable interaction performance in unknown environments.

While the experimental results confirm the effectiveness of the proposed method, the current study mainly focuses on a representative set of contact scenarios and on one-dimensional normal-direction control, so as to enable a clear and controlled validation of the proposed adaptive damping strategy. The real-robot experiments provide meaningful practical verification, and the main additional online computational cost is limited to policy-network inference, while the training process is performed offline in simulation, which supports real-time implementation. Future work will extend the method to broader task settings, including a wider range of materials, geometries, and contact conditions, as well as multi-axis and hybrid force/motion control problems. Further efforts will also address issues relevant to industrial applications, such as sim-to-real robustness, sensing uncertainty, actuator constraints, and safety requirements.

## Figures and Tables

**Table 1:** Final training performance statistics of different methods.

Method	Final Mean ± SD	Best Return	Conv. Seeds
Pure DDPG	1462.3 ± 58.4	1581.7 ± 73.9	8/10
DDPG + HER	352.1 ± 171.6	1087.5 ± 296.2	4/10
DDPG + TO-HER	1768.4 ± 67.9	1889.1 ± 61.4	8/10
DDPG + TO-HER + IS	2215.7 ± 52.6	2318.6 ± 49.8	10/10

**Table 2:** Experimental contact conditions and reference-force settings.

Surface Profile	Geometry	Reference Force	Motion Speed	Trajectory Length
Flat + circular-arc ramp	Ramp length: 200 mm; height: 40 mm	Constant 20 N	10 mm/s	300 mm
Flat + inclined plane	Slope length: 200 mm; height: 40 mm	Constant 20 N	10 mm/s	300 mm
Fully flat surface	Flat over the entire path	20 N (0–10 s), 40 N (10–20 s), 20 N (20–30 s)	10 mm/s	300 mm

Notes: For all cases, the contact material was polished aluminum alloy with a relatively smooth surface finish.

**Table 3:** Force-Tracking Performance under Different Contact Environments.

Environment	Algorithm	Mean Err. (N)	RMSE (N)	Overshoot (%)
Curved surface	Proposed	0.516 ± 0.042	0.868 ± 0.071	26.32 ± 2.84
	Adaptive Impedance	1.735 ± 0.216	2.847 ± 0.391	67.27 ± 43.15
	Constant Impedance	11.243 ± 1.384	13.712 ± 1.695	109.93 ± 12.74
Slope surface	Proposed	0.311 ± 0.036	0.745 ± 0.062	29.57 ± 2.63
	Adaptive Impedance	0.678 ± 0.089	1.380 ± 0.174	45.51 ± 5.42
	Constant Impedance	9.680 ± 1.127	11.939 ± 1.451	114.82 ± 33.68
Varying force reference	Proposed	0.614 ± 0.058	1.553 ± 0.126	31.06 ± 2.73
	Adaptive Impedance	0.675 ± 0.073	1.964 ± 0.203	45.51 ± 4.58
	Constant Impedance	1.629 ± 0.184	3.022 ± 0.317	60.99 ± 6.12

Note: Values are reported as mean ± SD over five trials.

## Data Availability

The original contributions presented in this study are included in the article. Further inquiries can be directed to the corresponding author.
